# Thymoquinone improves cognitive and hippocampal long-term potentiation deficits due to hepatic encephalopathy in rats

**DOI:** 10.22038/ijbms.2021.52824.11913

**Published:** 2021-07

**Authors:** Somayeh Hajipour, Yaghoob Farbood, Mahin Dianat, Mohammad Rashno, Laya Sadat Khorsandi, Alireza Sarkaki

**Affiliations:** 1 Persian Gulf Physiology Research Center. Medical Basic Sciences Research Institute, Ahvaz Jundishapur University of Medical Sciences, Ahvaz, Iran; 2 Department of Physiology, Faculty of Medicine, Ahvaz Jundishapur University of Medical Sciences, Ahvaz, Iran; 3 Department of Immunology, Cellular & Molecular Research Center, Medical Basic Sciences Research Institute, Ahvaz Jundishapur University of; 4 Medical Sciences, Ahvaz, Iran; 5 Department of Anatomical Sciences, Cellular & Molecular Research Center, Medical Basic Sciences Research Institute, Ahvaz Jundishapur University; 6 of Medical Sciences, Ahvaz, Iran; 7 Medicinal Plant Research Center, Ahvaz Jundishapur University of Medical Sciences, Ahvaz, Iran

**Keywords:** Hepatic encephalopathy, LTP, Oxidative stress, Spatial cognition, Thioacetamide, Thymoquinone

## Abstract

**Objective(s):**

Hepatic encephalopathy (HE) is a neuropsychiatric syndrome that causes brain disturbances. Thymoquinone (TQ) has a wide spectrum of activities such as antioxidant, anti-inflammatory, and anticancer. This study aimed to evaluate the effects of TQ on spatial memory and hippocampal long-term potentiation (LTP) in rats with thioacetamide (TAA)-induced liver injury and hepatic encephalopathy.

**Materials and Methods:**

Adult male Wistar rats were divided into six groups randomly: 1) Control; 2) HE, received TAA (200 mg/kg); 3-5) Treated groups (HE+TQ5, HE+TQ10, and HE+TQ20). TQ (5, 10, and 20 mg/kg) was injected intraperitoneally (IP) for 12 consecutive days from day 18 to 29. Subsequently, spatial memory performance was evaluated by the Morris water maze paradigm and hippocampal LTP was recorded from the dentate gyrus (DG) region. Activity levels of Malondialdehyde (MDA) and superoxide dismutase (SOD) were measured in the hippocampal tissue.

**Results:**

Data showed that the hippocampal content of MDA was increased while SOD activities were decreased in TAA-induced HE. TQ treatment significantly improved spatial memory and LTP. Moreover, TQ restored the levels of MDA and SOD activities in the hippocampal tissue in HE rats.

**Conclusion:**

Our data confirm that TQ could attenuate cognitive impairment and improve LTP deficit by modulating the oxidative stress parameters in this model of HE, which leads to impairment of spatial cognition and LTP deficit. Thus, these results suggest that TQ may be a promising agent with positive therapeutic effects against liver failure and HE defects.

## Introduction

Hepatic encephalopathy (HE) is a neurological complication of the liver, which can lead to reduced quality of life and more severe outcomes in cirrhotic patients (1, 2), and it is identified by cognitive deficits, personality changes, and reduced attention (3). These neuropsychiatric alterations may finally lead to coma and death (4).

Although the pathophysiology of HE is not completely understood, some theories have suggested that overproduction of ammonia during liver damage plays a key role in HE (1, 5). Ammonia is a product of degradation of proteins and other nitrogenated combinations; high concentrations of ammonia and other toxic substances can reach the brain and alter its functions (1). Alterations in cognitive functions have been proven in different models of acute and chronic HE, showing certain decreases in performance in tasks evaluating learning capability and spatial memory (6, 7).

Several studies have shown that spatial learning and memory were impaired in different experimental models of liver failure such as bile duct ligation (BDL) (8), portacaval shunt (6, 7, 9, 10), and thioacetamide intoxication (7). Studies showed that impaired LTP in the hippocampus could contribute to some of the alterations in learning and memory in hyperammonemia and HE (11). 

Long-term potentiation (LTP) is a type of synaptic plasticity that is necessary for storage of information in the hippocampus, which plays an important role in learning and memory processes (10). LTP induction requires the activation of glutamate receptors, while hyperammonemia and liver failure bring about changes in the glutamatergic neurotransmission that can impair LTP and cognitive function in patients with liver disease (12-14). 

An experimental rat model of HE is induced by administration of thioacetamide (TAA). This model is very comparable to human cases that have progressive hepatic disorders with parallel involvement of the brain (15, 16). TAA is metabolized to extremely reactive metabolites such as thioacetamide sulfine and sulfene. These compounds can permanently bind to various macromolecules in hepatocytes (17), resulting in hepatic necrosis (18), hyperammonemia (18-21), and widespread oxidative stress (21, 22). Several studies have shown that TAA produces a large amount of reactive oxygen species (ROS), which can suppress the anti-oxidant defense mechanism and damage cellular components such as lipids, proteins, and DNA. This, in turn, can impair cellular structure and function (22-24).

Thymoquinone (TQ) (2-isopropyl-5-methyl-1, 4-benzoquinone) is a bioactive compound derived from *Nigella sativa* that is frequently used in some Middle Eastern and Far Eastern countries for the purpose of prevention and treatment of a large number of diseases (25-27). TQ has various pharmacological properties, including antihypertensive, anticancer, antidiabetic, and anti-inflammatory (28-30). Studies have shown that administration of TQ is not accompanied by toxic side effects in the experimental models (31). A study reported that the hydro-alcoholic extract of *N. sativa *provided protection against synaptic plasticity as well as spatial learning and memory impairments induced by lipopolysaccharide (LPS) in rats (25). Furthermore, TQ offers protective effects against acetaminophen-induced hepatotoxicity by attenuating free radical-induced oxidative injuries, as well as protection against cyclophosphamide-induced cardiotoxicity and bile duct ligation-induced liver damage in animal models (32-34). On the other hand, it has been proposed that TQ may act as an anti-oxidant agent, scavenger of superoxide, hydroxyl radical, and might prevent membrane lipid peroxidation in hepatocytes (35, 36). 

Therefore, this study aimed to evaluate the therapeutic effects of TQ on spatial cognition, synaptic field potential deficits in the hippocampus, and oxidative damage of the brain tissue in a rat model of TAA-induced HE.

## Materials and Methods

***Animals***

Sixty adult male Wistar rats (three months old, weighing 200–250 g) were obtained from the main animal care and breeding center of Ahvaz Jundishapur University of Medical Sciences (AJUMS). All animals were housed in standard cages under controlled temperature (22 ± 2 °C), humidity (50–55%), and a 12 hr light/dark cycle (lights on at 07:00 am), with *ad libitum* access to food chow pellets and tap water. All experimental protocols were approved by the Ethics Committee of AJUMS (Ethic code: IR.AJUMS.REC1396.684).

***Preparation of drugs***

TAA and TQ were purchased from Sigma (St. Louis, MO, USA). TAA was prepared freshly by dissolving it in sterile distilled water and TQ was dissolved in sterile distilled water containing 5% DMSO.

***Experimental protocols***

After a three-day period of handling for acclimatization, the rats were divided randomly into six groups (with 10 in each):

1) Control: Rats received normal saline containing 5% DMSO (2 ml/kg, IP) as vehicle once every 48 hr for 14 consecutive days.

2) HE: rats received TAA (200 mg/kg/2 ml, IP) once every 48 hr for 14 consecutive days for induction of the experimental rat model of HE and received the vehicle of TQ.

3) HE +TQ5: HE rats received TQ (5 mg/kg, IP) once daily for 12 consecutive days from 24 hr after the last injection of TAA.

4) HE +TQ10: HE rats received TQ (10 mg/kg, IP) once daily for 12 consecutive days from 24 hr after the last injection of TAA.

5) HE +TQ20: HE rats received TQ (20 mg/kg, IP) once daily for 12 consecutive days from 24 hr after the last injection of TAA (37-39).

**Figure 1:**
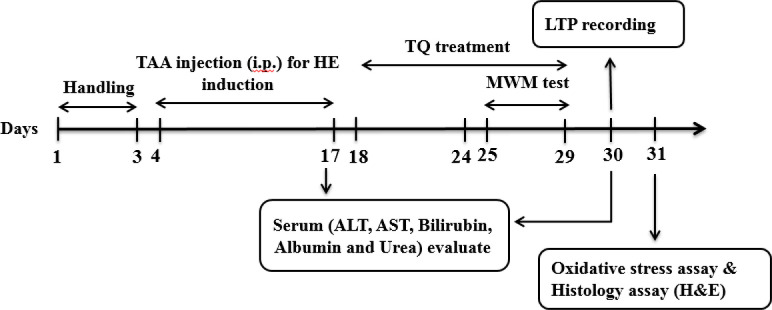
Timeline and design of experimental protocols

Each group was divided into two sub-groups for behavioral assessment followed by electrophysiological, biochemical, and histological evaluations of the liver. The timeline and experimental design of this work are presented in Figure 1.

***Induction of HE***

According to several lines of evidence, HE type A is caused by acute liver failure (ALF), and among the reasons behind the induction of this type are various toxins and drugs. In the current work, TAA was used to induce ALF, which in effect induced a model of acute HE or Type A. Since in some studies, fulminant hepatic failure (FHF) was induced by intraperitoneal injection of TAA (300 mg/kg) for three consecutive days (40, 41), which caused a high rate of mortality and did not allow the animals to live for a long time, therefore, several pilot experiments were conducted by the authors to explore and find an appropriate dose of TAA to induce ALF and allow the animals to live long enough in order to perform behavioral and electrophysiological tests. 

Finally, TAA-induced liver injury in this study was generated via injection of 200 mg/kg TAA once every 48 hr for 14 days to obtain both liver injury and development of hepatic encephalopathy. Control rats received normal saline containing 5% DMSO (2 ml/kg, IP). Twenty-four hours after the initial injection of TAA, all animals received 5% dextrose (25 ml/kg of body weight) daily containing 0.45% NaCl and potassium chloride (20 meq/l) intraperitoneally at 12 hr intervals to prevent hypoglycemia, weight loss, and renal failure as side effects of TAA. To establish liver failure, some related biofactors were measured such as serum total bilirubin, albumin, urea, and hepatic enzymes (ALT and AST).

***Morris water maze (MWM) test***

MWM is an established test for spatial cognition evaluation. This paradigm comprised an apparatus with a black circular pool (height 60 cm, diameter 150 cm) that was filled with water (24 ± 1 °C) to a depth of 40 cm. The pool was divided into four equal quadrants including north (N), east (E), south (S), and west (W). A black escape platform (10 cm in diameter) was placed in one of the four quadrants and was submerged 1.5 cm below the water surface, and thus it was invisible. A digital camera was mounted 200 cm above the center of the maze in order to track the animals’ swimming paths. The escape latencies and percentage of the time spent in the target quadrant during the probe trial were measured by a video-tracking software (Ethvision software Ver. 7, Noldus Co., Netherlands). All rats had four trials per session in four consecutive days of training. During the experiments, each animal was randomly placed in one quadrant (N, E, S, or W) and was allowed to freely swim for 60 sec to find the hidden platform and then rest for 30 sec until the next trial. Twenty-four hours after the last learning trial (on the 5^th^ day), a probe trial was conducted by removing the hidden platform in order to evaluate spatial memory. During this trial, the rats were allowed to swim freely for 60 sec. The time spent in the target quadrant (where the platform was placed during the acquisition phase) was recorded as memory retrieval (42, 43).

***Electrophysiological evaluation***

The hippocampal long-term potentiation (LTP) recording was carried out from the dentate gyrus (DG) area of a few rats (n = 5). The rats were anesthetized with urethane (1.5 g/kg, IP) and their heads were positioned on a stereotaxic apparatus (SR-6 N; Narishige, Japan). Then the skulls of the rats were drilled where the stimulating and recording electrodes would be implanted. A bipolar stimulating metal wire microelectrode (stainless steel, 100 μm in diameter, tip separation 500 μm, CFW, USA) was placed into the perforant pathway (PP) at AP: - 7.5 mm from bregma; ML: -4 mm; DV: 3.9 mm from the dura, and a metal wire recording microelectrode (tungsten, 50 μm in diameter, tip separation 1 mm, CFW, USA) was implanted on the granular cells of the dentate gyrus (DG) of the left hemisphere with the following coordinates: AP; -3.8 mm to bregma, ML; -2.3 mm, DV; 3.5 mm from skull surface according to the atlas of Paxinos and Watson (44). In order to induce LTP, the PP was stimulated by single monopolar pulses (length 50 µ sec) at 30-sec intervals and the different field potential recordings were observed in the DG area (45). The post-tetanic stimulation population spike (PS) amplitude was obtained as the difference in voltage between the peak of the first positive wave and the peak of the first negative deflection. The maximum slope between the initial point of fEPSP and the first positive peak of the wave was quantified as the fEPSP slope in order to measure synaptic efficacy. The field excitatory postsynaptic potential (fEPSP) with 40% of its maximum amplitude was selected as baseline intensity, and high-frequency stimulation (HFS) with 80% of its maximum amplitude was selected as tetanic stimulation shown by an input/output (I/O) curve (46). Extracellular field potentials were amplified (100×), filtered (0.1 Hz–3 kHz), and analyzed using eProbe software (version 1.53, Science Beam Co., Iran). LTP was recorded for periods of 5, 15, 30, 45, and 120 min after HFS in order to determine any alterations in the synaptic response of DG neurons (47).

***Serum biochemical analysis***

Serum biomarkers were measured in all the tested groups during two stages: at the end of TAA administration (17^th^ day) and the end of the treatment of the HE rats with different doses of TQ (31^th^ day). Blood samples were collected from the left ventricle of the hearts and were centrifuged (3000×g, 4 °C, 20 min) to separate the serum. All serum biomarkers were determined using reagent kits. Serum levels of aspartate aminotransferase (AST), alanine aminotransferase (ALT), albumin, total bilirubin, and urea were determined calorimetrically using commercial kits.

***Biochemical measurements***

*Preparation of brain homogenates*

Rats were deeply and irreversibly anesthetized with sodium pentobarbital (90 mg/kg, IP). Afterward, the animals were decapitated and their brains were removed from the skulls; after which, hippocampal tissues were quickly removed on ice, cleaned with saline, and frozen at -80 °C. In the next step, the hippocampal tissues were homogenized in a cold phosphate buffer saline (PBS), and then the samples were centrifuged at 10,000 rpm for 20 min at 4 °C, and the supernatant was collected for direct assessment of biochemical parameters. Bio-Rad protein assay kit was used according to the manufacturer’s protocols to calculate the total protein concentration of each sample. The values of the hippocampus content of SOD and MDA were analyzed by ELISA kits and the acquired data were calculated as nmol/mg of the supernatant (48).

*Measurement of hippocampal MDA level*

An ELISA kit (ZellBio GmbH, Cat. No: ZB-MDA-96A, Germany) was used to measure the malondialdehyde (MDA) produced in the brain tissue. The assay was performed according to the manufacturer’s guidelines. MDA is a product of lipid peroxidation produced by direct damage of oxidative toxicity to cellular polyunsaturated fatty acids (PUFA). The level of MDA, an index for lipid peroxidation, was determined by thiobarbituric acid reactive substances (TBARs) assay. Results are reported as nmol of MDA per milligram of protein (nmol/mg protein) (49).

*Measuring superoxide dismutase (SOD) activity in the hippocampus*

SOD activity in hippocampal tissue was evaluated by an ELISA kit (ZellBio GmbH, Cat. No: ZB-SOD-96A, Germany). In this procedure, xanthine and xanthine oxidase are used to make superoxide radicals, which react with 2-(4-iodophenyl)-3-(4-nitrophenol)-5phenyltetrazolium chloride (INT) to form a red formazan color. SOD activity in the sample was defined by the inhibition degree of this reaction and is presented as units/mg of protein (50).

***Histological assay***

After blood collection, the rats’ livers were removed immediately and fixed in a 10% formalin solution. In the next step, they were dehydrated in graded alcohol concentrations and then embedded in paraffin. Four sections of 6 µm thickness were prepared and stained with hematoxylin and eosin (H&E). Six microscopic slides per animal were examined for assessment of histological changes such as accumulation of RBCs, infiltration of inflammatory cells, fat deposit in hepatocytes, and necrosis. The histological features were graded into four categories: normal (0), weak (1), moderate (2), and severe (3). For each slide, the mean of six fields was calculated and the slides were read in a “blind” fashion.

***Statistical analysis***

The results were presented as mean ± SEM and the data normality was assessed using the Kolmogorov-Smirnov test. The MWM test and LTP’s data were analyzed using repeated measures one-way ANOVA followed by Tukey’s *post hoc* test. Other data were analyzed by one-way ANOVA followed by Tukey’s *post hoc* test. *P*<0.05 was considered as a statistically significant difference between experimental groups. All statistical analyses were done using GraphPad Prism software (version 6, GraphPad Software Inc., San Diego, USA).

## Results

***Effects of TQ on spatial learning and memory***

**Figure 2:**
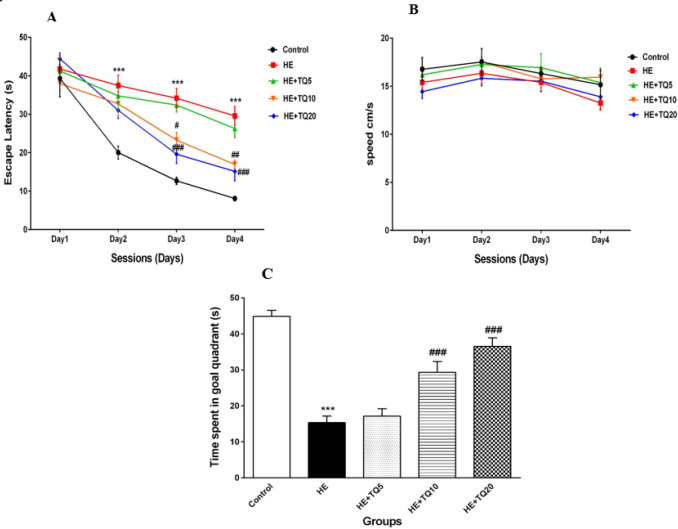
Spatial learning and memory in Morris water maze. (A) Mean escape latency to reach the hidden platform evaluated in all rats for four consecutive days. (B) Swimming speed. (C) Probe trial test for spatial memory retrieval. Data were analyzed by repeated-measures two-way ANOVA, followed by Tukey's* post-hoc* test. HE: Hepatic encephalopathy, TQ: Thymoquinone. *** *P*<0.001 vs control group, # *P*<0.05, ## *P*<0.01, and ### *P*<0.001 vs HE group

After four days of training in MWM, the results showed that the escape latency to find the hidden platform was significantly higher in the HE group in contrast to the control group during the second to fourth training days (*P*<0.001); while treatment with TQ for seven consecutive days, significantly decreased the escape latency in the HE+TQ10 and HE+TQ20 groups compared with the HE group (*P*<0.01 and *P*<0.001, respectively, Figure 2A). However, there were not any significant changes between HE+TQ5 and HE groups in any of the training days (*P*>0.05 for each comparison). 

Furthermore, results showed no significant difference in swimming speed among any of the tested groups (*P*>0.05; Figure 2B).

As illustrated in Figure 2C, the results of the probe trial concerning evaluating the memory retrieval 24 hr after the last trial, showed that the rats in the HE group spent less time in the target quadrant compared with the control group (*P*<0.001). However, treatment with TQ (10 and 20 mg/kg) significantly increased the percentage of time spent in the target quadrant when compared with the HE group (*P*<0.001 and *P*<0.001, respectively). There was no significant difference in the time spent in the target quadrant between HE+ TQ5 and HE groups (*P*>0.05). 

***Hippocampal LTP***

**Figure 3:**
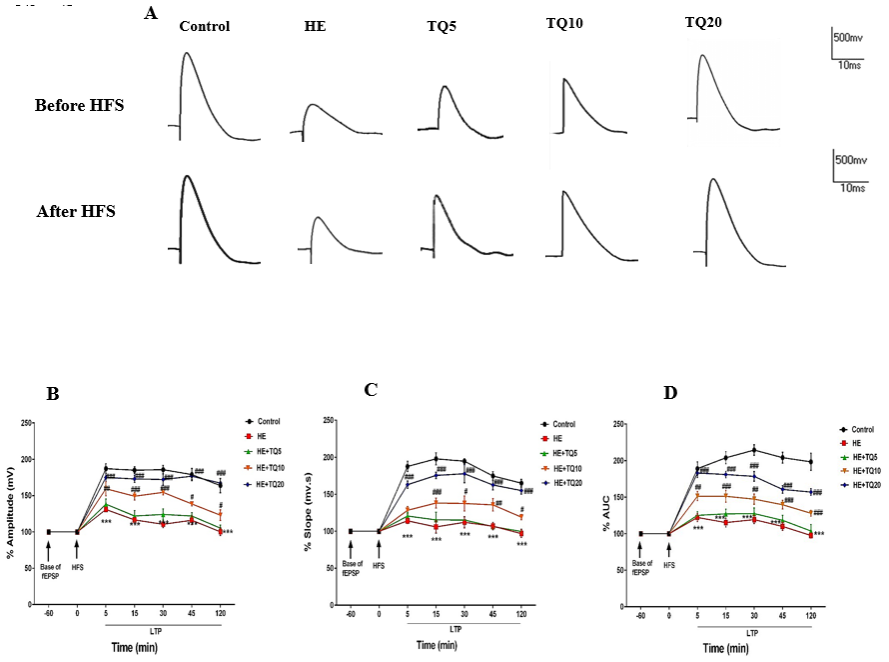
LTP recordings after high-frequency stimulation (HFS) to the perforant path (PP) in different groups. (A) Sample traces recorded from the hippocampal DG area. The upper panel shows the records before HFS while the lower panel shows the records after HFS to PP. (B) Percentages of population spike amplitude, (C) percentages of fEPSP slope, and (D) percentages of the area under the curve. Data were analyzed by repeated-measures two-way ANOVA followed by Tukey's *post-hoc* test. Data are presented as mean ± SEM, (n=5). HE: Hepatic encephalopathy, TQ: Thymoquinone. *** *P*<0.001 vs control group, # *P*<0.05, ## *P*<0.01, and ### *P*<0.001 vs HE group

Sample traces recorded from the hippocampal DG area of all tested groups in both states of before and after high-frequency stimulation (HFS) are shown in Figure 3A.

PS Amp: As demonstrated in Figure 3B, the amplitude (mv) of population spike (PS) was decreased significantly (*P*<0.001) in the HE group during all LTP recordings after HFS in comparison with the control group; while it was significantly increased in similar points in time in the groups treated with TQ (doses of 10 and 20 mg/kg) when compared with the HE group (*P*<0.05 and *P*<0.001, respectively). Furthermore, treatment of HE rats with TQ in the HE+TQ5 group did not aﬀect PS amplitude as compared with the HE group (*P*>0.05). 

fEPSP slope: As shown in Figure 3C, the fEPSP slope was significantly decreased in the HE rats in comparison with the control group (*P*<0.001) in different points in time, while it was significantly improved in TQ10 and TQ20 groups (*P*<0.05 and *P*<0.001, respectively). However, treatment with TQ in the HE+TQ5 group did not aﬀect fEPSP slope as compared with the HE group (*P*>0.05).

PS AUC: As illustrated in Figure 3D, the results of two-way repeated-measures ANOVA analysis showed that AUC was significantly reduced at 5, 15, 30, 45, and 120 min after HFS recording in the HE group compared with the control group (*P*<0.001); whereas AUC of PS in TQ-treated groups (TQ10 and TQ20) was significantly higher than in the HE group (*P*<0.001 and *P*<0.001, respectively) in the same recording periods. Nevertheless, data showed that there were not any significant changes in AUC between HE+TQ5 and HE groups (*P*>0.05).

***Serum biochemical indexes***

The results revealed that induction of HE by TAA caused a significant increase in serum ALT versus the control group (*P*<0.001). Treating HE rats with TQ (10 and 20 mg/kg) for seven consecutive days caused a significant decrease in serum ALT level in comparison with the HE group (*P*<0.01 and *P*<0.001, respectively). TQ at the dose of 5 mg/kg in the HE+TQ5 group did not cause any significant decrease in the serum ALT content compared with the HE group (*P*>0.05).

**Figure 4:**
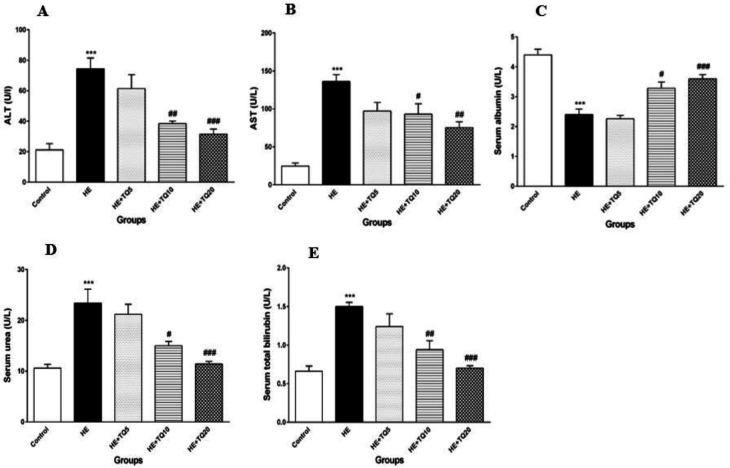
Effects of TQ treatment on biochemical indexes in serum in different experimental groups. (A and B) serum ALT and AST levels, respectively; (C) serum albumin concentration; (D) serum urea levels; (E) serum total bilirubin concentration. Data are presented as mean ± SEM (n = 6). HE: Hepatic encephalopathy, TQ: Thymoquinone. ***P<0.001 vs control group, # *P*<0.05, ## *P*<0.01, and ### *P*<0.001 vs HE. Data were analyzed statistically by one-way ANOVA followed by Tukey's* post-hoc* test

Data showed that serum AST level was significantly increased in the HE rats compared

with the control group (*P*<0.001), while it was significantly decreased in both TQ-treated groups (HE + TQ10 and HE + TQ20) compared with the HE group (*P*<0.05 and *P*<0.01, respectively; Figure 4B). Furthermore, at the dose of 5 mg/kg in the HE+TQ5 group, TQ did not cause any significant decrease in the serum AST level compared with the HE group (*P*>0.05).

As it can be observed in Figure 4C, serum level of albumin was significantly decreased in the HE group (*P*<0.001), when compared with the control rats. Serum albumin level was significantly raised in the HE rats that were treated with TQ10 and TQ20 (*P*<0.05 and *P*<0.001, respectively). However, treatment with 5 mg/kg TQ in the HE+TQ5 group did not cause any significant increase in the serum level of albumin compared with the HE group (*P*>0.05).

The level of serum urea was increased significantly in the HE rats after TAA administration in comparison with the control group (*P*<0.001). Administration of TQ (10 and 20 mg/kg) significantly reduced the serum urea level in the HE rats (*P*<0.05 and *P*<0.001, respectively) compared with the HE group. Whereas, data showed that there were not any significant changes in the serum urea levels in HE+ TQ5 and HE groups (*P*>0.05) (Figure 4D).

As shown in Figure 4E, HE rats showed significantly higher serum total bilirubin in contrast to the control group (*P*<0.001). Treating HE rats with TQ (10 and 20 mg/kg) significantly reduced the level of total bilirubin in serum compared with the HE group (*P*<0.01 and *P*<0.001, respectively). At the dose of 5 mg/kg in the HE+TQ5 group, TQ did not cause

any significant decrease in the serum total bilirubin level compared with the HE group (*P*>0.05). 

***Oxidative stress***

*Lipid peroxidation (MDA level)*

**Figure 5:**
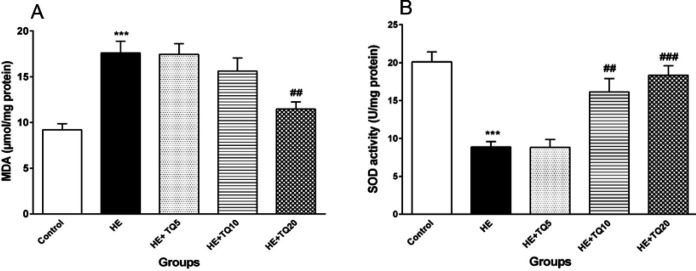
Effects of TQ on the levels of malondialdehyde (MDA) in hippocampal tissue and superoxide dismutase (SOD) in TAA-induced HE rats. (A) Hippocampal tissue level of MDA; (B) levels of SOD activity in hippocampal tissue in all the groups. Data are presented as mean ± SEM (n = 5). HE: hepatic encephalopathy group, TQ: thymoquinone. *** *P*<0.001 vs control group, ## *P*<0.01 and ### *P*<0.001 vs HE group. Data were analyzed by one-way ANOVA followed by Tukey's *post-hoc* test

The results confirmed that the MDA level was significantly higher in the hippocampal tissue of the HE group compared with the control group (*P*<0.001); while the MDA level in the hippocampal tissue of the HE rats treated with TQ20 was significantly decreased in comparison with the HE group (*P*<0.01). In contrast, doses of 5 and 10 mg/kg of TQ did not induce any significant reduction in the MDA level (Figure 5A).

*Superoxide dismutase (SOD) activity*

As shown in Figure 5B, there was a significant decrease in SOD activity in the hippocampal tissue of the animals in the HE group in comparison with the control rats (*P*<0.001). SOD activities of hippocampal tissues in the groups treated with doses of 10 and 20 mg/kg of TQ were increased significantly compared with the HE group (*P*<0.01 and *P*<0.001, respectively). However, data showed that there were not any significant changes in the hippocampal level of SOD activity in HE+ TQ5 and HE groups (*P*>0.05).

***Liver histopathology***

**Figure 6:**
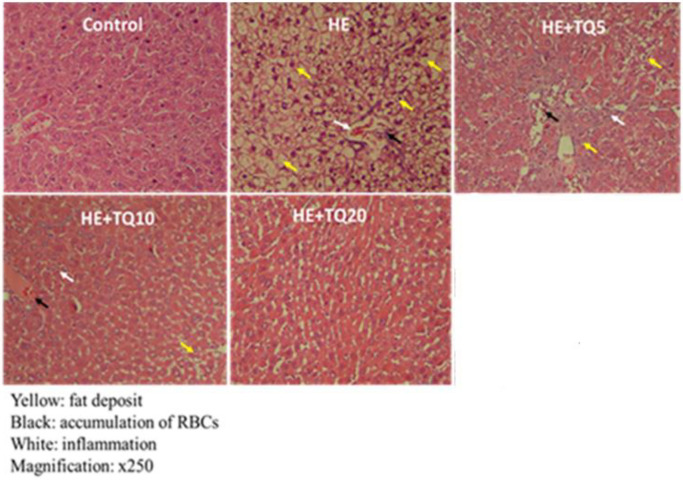
Histopathological analyses for liver tissues. Images are taken from livers and the results of H&E staining in the animals

Histological findings showed that administration of 200 mg/kg of TAA had resulted in induction of widespread necrosis and inflammation in hepatic lobules, as well as mild to moderate portal inflammations. After administration of TQ (5, 10, and 20 mg/kg), minimal portal inflammation and absence of hepatocyte apoptosis were observed (Figure 6A). Therefore, histological findings indicated that TQ could effectively reduce inflammation and necrosis in hepatic tissue in a dose-dependent manner (Table 1).

**Table 1:** Quantitative data of the results of hepatic histology in different tested groups. Values of each bar are expressed as mean ± SEM

Groups	RBCs accumulation	Inflammation	Fat deposit (%)
Control	0.02±0.00	0.03±0.00	0.00±0.00
HE	1.2±0.23^***^	1.4±0.29^***^	80.3±15.6^***^
HE+TQ5	1.3±0.14^***^	1.43±0.22^***^	68.9 ±5.3^***^
HE+TQ10	0.52±0.06^**#^	0.57±0.11^**##^	8.2±1.9^***###^
HE+TQ20	0.14± 0.06^**###^	0.08±0.05^*##^	1.9 ±0.62^***###^

HE: hepatic encephalopathy group, TQ: thymoquinone. * *P*<0.05, ** *P*<0.01, and *** *P*<0.001 vs control group, # *P*<0.05, ## *P*<0.01, and ### *P*<0.001 vs HE group

## Discussion

In the present study, it was demonstrated that induction of experimental acute HE caused spatial memory impairment and hippocampal long-term potentiation (LTP) deficit and disruption of some of the important serum biochemical, liver enzymes, and oxidative stress in the brain. The findings revealed that treatment with TQ improved spatial learning and memory, hippocampal LTP, and brain oxidative stress damage following HE induction, in a dose-dependent manner. 

HE is characterized by cognitive dysfunction (10, 51) and several reports have demonstrated learning impairment in different animal models of HE (6, 12). In this work, it was shown that treatment with TQ restores memory deficit, which was demonstrated by the increase in the time spent in the target quadrant during the probe trial test in the MWM task. Previous studies have indicated that *N. sativa* oil improves spatial cognitive functions in experimental rats (52, 53). Furthermore, the beneficial effects of *N. sativa* on human cognition, memory, and attention have also been confirmed (54). As a cellular mechanism of memory, LTP in the hippocampus plays a key role in some forms of learning and memory (10). Moreover, it has been reported that the lack of LTP induction in HE rats would be responsible for impairment of some cognitive functions, including reduced learning and memory in the Morris water maze (10, 11). Previous studies have shown that LTP is impaired in hippocampal slices (*in vitro* recording) obtained from experimental models of liver failure (55, 56). Spatial learning and memory and induction of LTP are modulated through AMPA and NMDA receptors in the hippocampus, and subsequent activation of glutamate-nitric oxide–cGMP pathway (57). During HE, the change in this modulation is associated with impaired LTP and consequently, reduced learning and memory (11). In this study, the LTP from the dentate gyrus (DG) region with pyramidal neurons was recorded by measuring the population spikes’ (PSs) amplitude, fEPSPs slope, and the area under the curve (AUC). The decreased PSs amplitude and fEPSPs slope in HE groups were consistent with the impaired LTP, which may account for their poor performance in the MWM test. The present research provides new findings as treatment of HE rats with TQ ameliorated synaptic plasticity deficit in the DG region of the hippocampus. These results showed that TQ treatment could increase fEPSP slope, PS amplitude, and AUC following high-frequency stimulation (HFS) in HE rats. Having mentioned that, the mechanism(s) responsible for the effects of TQ on LTP has not been investigated. Nevertheless, researchers showed that *N. sativa* can increase glutamate release and affect AMPA receptors, which may be considered as possible mechanisms for the positive effects of *N. sativa* on LTP (25). Rendeiro *et al*. (2009) reported that *N. sativa* seeds can induce learning and memory processes through increasing synaptic plasticity and long-term potentiation (58). 

Several studies have shown that treatment with TAA leads to oxidative stress and increase in several serum enzymes of the liver such as AST and ALT, which occur due to liver cell necrosis that eventually leads to the release of these enzymes into the blood circulation (59). Al-Attar (2011) reported a rise in serum lipid triglyceride and liver enzymes, and a reduction in serum total protein in HE animal models (60). 

The obtained data showed increases in bilirubin and urea content in the HE group that were accompanied by elevated serum activities of liver enzymes (ALT and AST) in comparison with the control group. Exposure to TAA resulted in a diminished number of functional hepatocytes. These findings are supported by disturbed histopathological images showing features of degeneration, necrosis, and inflammation in the livers of HE rats. In this condition, the liver loses its ability to detoxify toxic substances such as ammonia from the blood.

 As shown in the present study, TQ treatment significantly diminished serum bilirubin and urea concentration, decreased the activities of serum ALT and AST, and significantly increased serum albumin content compared with the HE group (Figure 4). The histopathological picture of liver tissues of the rats treated with TQ supported this data by the improvements observed in the overall histopathological pictures (Figure 6 and Table 1). In concordance with this study, A study (2012) showed that oral administration of TQ maintained anti-oxidant defenses and reduced liver oxidative damage in bile duct ligated rats (61).

Additionally, TAA-induced HE in the current work significantly increased the MDA level and decreased SOD in the hippocampal tissue in HE rats, which may have resulted from the elevated oxidative stress. Several studies have shown that TAA administration induces liver failure, while potentially damaging the brain (16, 62-64). Besides, there are reports indicating that using TAA to induce experimental HE, causes generation of free radicals and oxidative stress (62), and it has been confirmed that free radical-induced oxidative stress has a prominent role in the pathogenesis of HE (65). Evidence has shown that administration of TAA reduces the anti-oxidant level and increases lipid peroxidation in liver and brain tissues in experimental HE models (66). Reddy *et al*. (2004) and Fadillioglu *et al*. (2010) demonstrated the existence of different oxidants and anti-oxidants in the brain tissue following TAA-induced HE in rats. They also reported a significant reduction in anti-oxidant enzyme activities and increases in protein oxidation and lipid peroxidation produced in different parts of the brain (19, 67). Since the brain is very susceptible to oxidative stress (68), pathologically-induced oxidative stress could cause brain dysfunction and result in impaired learning and memory (69, 70). Furthermore, some other evidence supports the concept of ROS and their involvement in the oxidative pathway of memory impairment (71). In the present study, it was demonstrated that TQ reduces the lipid peroxidation caused by ROS and increases the anti-oxidant enzyme activities (SOD) following HE induction. Similar studies have shown the protective effect of TQ in carbon tetrachloride-induced hepatotoxicity through prevention of the lipid peroxidation process (72). Furthermore, several reports have indicated that TQ behaves as an effective anti-oxidant and increases the activity of several enzymes such as superoxide dismutase (SOD), glutathione transferase, and glutathione (GSH) (35, 39). 

In accordance with the previous studies, two main factors play a role in the pathogenesis of HE, including oxidative damage and increased blood ammonium (73). On the other hand, hyperammonemia decreases glutamate uptake and enhances extracellular glutamate levels leading to activation of N-methyl-D-aspartic acid (NMDA) receptor in the brain cortex resulting in elevated intracellular calcium (Ca^2+^) and increased generation of cyclic guanosine monophosphate (cGMP), and these events lead to alterations in a number of biochemical functions in the brain, including induction of nitrosative and oxidative stress as important factors in the pathogenesis of HE (10). These changes support the idea that the effects of hyperammonemia on oxidative stress are mediated by activation of the NMDA receptor. These results are in line with other studies (74). Nagi *et al*. (2000) have shown that TQ possesses strong anti-oxidant properties and defends several organs against the oxidative damage induced by free radical-generating agents (36). It has also been observed that the hydro-alcoholic extract of *N. sativa* improves learning and memory in rats; and this quality has been ascribed to the protective effect of *N. sativa* and its compounds against the oxidative damage of the liver and brain tissues (53, 71, 75, 76). Another study has shown the anti-oxidant effects of TQ against lipopolysaccharide-induced hepatic toxicity, increased MDA concentrations, and apoptosis in rats (77). Consistently, these findings revealed that *N. sativa *oil has potential scavenging activities, it controls free radical production and serves as a neuroprotective herb by improving memory function in rats. In the current study, the pivotal role of TQ against development of acute HE and oxidative stress induced by TAA was reconfirmed in a rat model.

## Conclusion

In conclusion, the findings of this study suggest that TQ can prevent cognitive impairment and LTP deficits that occur due to ALF and thereby HE induction by TAA toxicity in rats. The reduced lipid peroxidation and increased anti-oxidative enzyme activity may be due to the reduction of oxidative stress. These observations suggest that TQ can be a promising therapeutic agent for restoring the brain complications caused by ALF and acute HE.
